# Depression and suicidal ideation among individuals with type-2 diabetes mellitus, a cross-sectional study from an urban slum area of Karachi, Pakistan

**DOI:** 10.3389/fpubh.2023.1135964

**Published:** 2023-02-23

**Authors:** Hina Sharif, Shah Sumaya Jan, Sana Sharif, Tooba Seemi, Hira Naeem, Zahida Jawed

**Affiliations:** ^1^Research and Publication Department, SINA Health and Education Welfare Trust, Karachi, Pakistan; ^2^Department of Anatomy, Government Medical College, Srinagar, Jammu and Kashmir, India; ^3^School of Public Health, University of Saskatchewan, Saskatoon, SK, Canada; ^4^SINA Health Education and Welfare Trust, Karachi, Pakistan

**Keywords:** depression, type 2 diabetes, chronic illness, slums population, low socio-economic background, long duration of illness, suicidal ideation

## Abstract

**Background:**

Suicidal thoughts and depression are associated with patients with diabetes, especially patients with low socioeconomic backgrounds and prolonged illness.

**Objective:**

We aimed to estimate suicidal thoughts and depression among patients with type 2 diabetes (T2D) in the slums of Karachi.

**Methods:**

This cross-sectional study was conducted across 38 locations in the slums of Karachi to understand depression, suicidal thoughts, and other supporting factors of depression associated with T2D. The three-item Oslo Social Support Scale, the Patient Health Questionnaire-9 (PHQ-9) scale, and the Ask Suicide Screening Questions were used to screen the patients.

**Results:**

A total of 504 study participants were interviewed, with a response rate of 98%. The prevalence of depression among patients with diabetes was 30.83%, and suicidal ideation was 20.39%. In the final multivariate analysis, being socioeconomically poor, physically disabled, and having poor social support were independent predictors of depression.

**Conclusion:**

Diabetes, low socioeconomic level, a lack of social support, and physical disability were all linked to depression. Therefore, trained health providers should conduct an early depression-focused routine screening for patients with diabetes.

## Introduction

Diabetes is considered one of the world's largest growing epidemics, and globally, many countries have declared it a public health emergency (1). As per the latest reports from the Centers for Disease Control and Prevention (CDC), ~26 million (8.3%) adults and children in the United States alone have type 1 diabetes (T1DM) or type 2 diabetes (T2DM). Undoubtedly, T2DM consists of most of the chunk of it. Around the globe, more than 347 million people have diabetes, with an estimated prevalence of 9.8% in men and slightly lower than 9.3% in women (2). By 2030, 438,000,000 individuals are anticipated to develop diabetes, making the disease more prevalent globally. In low- and middle-income countries, diabetes accounts for more than 70% of morbidity and 88% of deaths (3, 4). In 2002, the International Diabetes Federation estimated that 33,000,000 cases of diabetes existed in Pakistan alone, affecting 26.7% of Pakistani adults (5). This figure is exceptionally high and keeps adding year after year. There are also grounds to suspect that many people go undetected, which would significantly increase the prevalence and the likelihood of complications from untreated conditions.

*Diabetes* is a lifelong chronic disease that is psychologically distressing for a patient. A new diagnosis of diabetes is a significant burden in a patient's life. Many go through classic stages of grief, denial, anger, depression, and acceptance (6). Due to its persistent and significant burden placed on people with diabetes in terms of self-management of the disease, people with diabetes face a variety of consequences because of their long-term condition, including lifestyle changes and responses to long-term treatment, concerns about complications, need for continuous monitoring of glycemic control, associated disability, and symptoms that interfere with their daily life. Depressive disorder is one of the most common and overwhelming psychiatric disorders in people with diabetes. Studies have shown that depression is a common comorbidity in people with diabetes (7, 8). The prevalence of depression in patients with DM ranges from 7 to 84% compared to an estimated prevalence of only 3–4% in the general population (9, 10).

The presence of depression in patients with diabetes mellitus is associated with financial stress, poor general health due to associated comorbidities, and poor glycemic control. It also worsens diabetes prognosis, increases treatment non-compliance, reduces the quality of life, prolongs diabetes recovery, and increases mortality (11). Depression is also a significant risk factor for hospitalization and diabetes-related complications. Furthermore, such patients may experience psychological and psychosocial problems at the individual and interpersonal levels, all of which contribute significantly to and are correlated with depression and can lead to suicide in some circumstances. Location-specific prevalence and related characteristics exist in Pakistan. In regular hospitals, psychiatric aspects of chronic illnesses like diabetes are rarely considered. Most earlier investigations on the prevalence of depression and associated variables among people with diabetes were carried out in designated specialized diabetes care centers. This study aimed to determine the prevalence of depression and suicidal ideation in individuals with type 2 diabetes from an urban slum in Karachi, Pakistan.

The results of this study will help develop more effective programs to treat comorbid diabetes and depression. In addition, the study results will serve as a basis for other researchers aiming to conduct large-scale studies in slum areas of Pakistan. Those interested in exploring the relationship between depression, suicide, and type 2 diabetes will also benefit from this study.

## Methods

### Study area

A cross-sectional observational study of a disadvantaged community in Karachi, Pakistan, was conducted. From August 2022 through September 2022, we gathered and evaluated primary data from SINA Health, Education, and Welfare Trust (SINA) clinics. SINA is a non-profit organization assisting slum populations since 1998 through a network of 38 clinic facilities, including three mobile vans in slum regions. Each year, these clinics assist nearly one million individuals. They provide high-quality primary healthcare to the poor, particularly women, children, and adolescents.

### Study design and period

This was an institution-based cross-sectional study conducted from August to September 2022.

### Source and study population

The source population included all patients with diabetes being followed up in the outpatient department. The study population included all patients with diabetes seen for follow-up during the data-collecting period.

### Inclusion and exclusion criteria

All patients with diabetes older than 18 years who could communicate freely were enrolled. Those using antidepressant medications for depressed symptoms were eliminated since antidepressant medications might disguise depression signs and symptoms. Patients with DM who were newly diagnosed at the time of data collection were excluded from the research because adjustment disorder is more frequent in newly diagnosed patients than a full-blown depressive symptom. Finally, this study excluded diabetic individuals who were extremely ill.

### Study variables

Depression and suicidal ideation were the dependent variables. Independent variables included age, gender, marital status, ethnicity, religion, educational and occupational status, clinical factors (kind of diabetes, fasting blood sugar level, duration of diabetes, treatment type), and psychosocial factors (social support).

### Sample size determination

Since depression prevalence was not known in the local population, especially in slums, the anticipated prevalence of 50% was used to determine the sample size, with a 95% confidence level with 5% absolute precision and a 30% non-response rate. The sample size with that came out to be around 499.

### Sampling technique and procedure

At the outset of the study, all patients with diabetes who were being followed up in the outpatient department of SINA clinics were approached with the study's objectives. Initially, 504 patients expressed an interest in taking part in the study. However, four participants were initially eliminated because they needed to give written informed consent, and seven more were excluded because they needed to fulfill the inclusion criteria. The remaining 493, however, were included in the study.

### Method of data collection and tools

Face-to-face interviews were used to gather data using a pretested semi-structured questionnaire that included socio-demographic parameters, clinical features, the three-item Oslo Social Support Scale, the Patient Health Questionnaire-9 (PHQ-9) scale, and the Ask Suicide Screening Questions to screen suicidal ideation among those found with depression. The socio-demographic and clinical information was evaluated using questionnaires modified by studying related literature and the patient's medical records.

The initial stage was to collect demographic information and treatment regimen-specific questions from patients, such as age, gender, education, material status, income, residential location (urban or rural), ethnicity, diabetes history, length of disease, and smoking/tobacco usage.

The outcome variable (depression) was assessed using the PHQ-9 (12). It comprises nine items on a four-point Likert scale that assess each of the nine DSM-IV depression criteria. Patients were asked to recollect depressed symptoms within the previous 2 weeks, with answers ranging from 0 (not) to 3 (nearly every day). A PHQ-9 score of 5 indicated depression. PHQ-9 is the most extensively used depression screening instrument; the PHQ-9's final question tackles passive suicidal thoughts. The PHQ-9 is frequently utilized in primary care settings, where the clinic population has fewer patients who screen positive for suicide risk.

If a patient affirms passive ideation on the PHQ-9, a more extensive suicide risk assessment should be administered. In our study, we administered the Ask Suicide Screening Questions (ASQ) Toolkit developed by the National Institute of Mental Health (NIMH), a standardized suicide risk screening tool certified for medical patients aged eight and older. It consists of four yes/no screening questions, takes 20 s to administer, and provides a toolbox with safety tips, worksheets, scripts, brochures, and paths. Moreover, the amount of social support was determined by asking patients to rate the level of support they got from family and friends using the Oslo 3 social support scale. The scale was numbered from 3 to 14. Participants with scores of 3–8, 9–11, and 12–14 out of 14 were assessed to have poor, moderate, or high social support.

### Data quality assurance

To ensure uniformity, the questionnaire was translated from English to the local language (i.e., Urdu, Punjabi, Sindhi, Balochi, Pashto, and Kashmiri) and then back to English. Before data collection, the local questionnaire version was pretested on 5% of the total research participants with obtained Cronbach's alpha of 0.839. Based on the pretest results, a slight change was made to the questionnaire's content. In addition, the questionnaire content and data-collecting techniques were taught to data collectors.

### Data processing and analysis

All data obtained were reviewed for completeness and consistency before being input into Microsoft Excel 2007 and exported to SPSS version 23 for analysis. To describe the socio-demographic features, clinical variables, and depression, descriptive statistics (frequencies, tables, percentages, and averages) were generated. Bivariate and multivariate logistic regression analyses were performed. To avoid possible confounders, variables with *p*-values < 0.05 in the bivariate model were added to the multivariate analysis. Variables with *p*-values of < 0.05 were deemed statistical predictors of depression in the multivariate model. The strength of the link was measured using the odds ratio with a 95% confidence interval.

## Results

A total of 504 participants were approached for the study with a 2% non-response rate, so 493 participants fulfilling the inclusion criteria were finally taken up for final analysis. Table 1 shows the sociodemographic analysis of the studied participants. The mean age of participants was 48.3 (SD± 12.8) years. Maximum participants were female (78.9%), in the age group of 46–55 years (34.5%), were illiterate (70.0%), married (96.0%), had no source of income (81.5%), lived in urban setup (94.8%), has no employment (81.5%), were Urdu speaking (20.4%), has a family history of diabetes mellitus (70.4%), were non-regular to daily exercise regimen (67.2%), no history of smoking (81.1%).

**Table 1:** Socio-demographic characteristics of patients with diabetes mellitus attending the outpatient department of SINA Clinics [August–September 2022] (*N* = 493).

**Demographic characteristics**	**Co-variables**	***n* (%)**
Sex	Male	104 (21.1)
	Female	389 (78.9)
Age groups	18–24	3 (0.6)
	25–35	21 (4.2)
	36–45	106 (21.5)
	46–55	174 (34.5)
	56–65	123 (24.9)
	≥66	63 (13.2)
Education	Illiterate	344 (70.0)
	Primary	55 (11.4)
	Secondary	60 (12.2)
	Intermediate	18 (3.7)
	Graduate	7 (1.4)
	Postgraduate	6 (1.4)
Marital status	Married	484 (96.0)
	Unmarried	6 (1.2)
Income	No income	411 (81.5)
	< 13,000	28 (5.5)
	13,001–39,000	41 (8.13)
	39,001–64,000	3 (0.6)
	64,001 and above	7 (1.38)
Residential location	Urban	478 (94.8)
	Rural	12 (2.3)
Employment	Yes	77 (15.2)
	No	411 (81.5)
Ethnicity	Urdu speaking	103 (20.4)
	Punjabi	50 (10.1)
	Sindhi	33 (6.1)
	Baloch	12 (2.3)
	Abbottabad	9 (1.7)
	Bengali	16 (3.17)
	Hazara	1 (0.2)
	Pathan	208 (41)
	Saraiki	11 (2.1)
	Hindko	11 (2.2)
	Kashmiri	11 (2.2)
Diabetes family history	Yes	355 (70.4)
	No	151 (26.6)
Exercise	Yes	152 (30.8)
	No	339 (67.2)
Smoking	Yes	81 (16.0)
	No	409 (81.1)

Of the surveyed participants, (37.7%) were diagnosed when they were more than 55 years of age, while (35.2%) of participants were diagnosed between ages 45–54. The majority (35.9%) were suffering from the illness for 1–5 years duration, (58.3%) were on oral hypoglycemics agents (OHA) while (and 37.5%) were using both OHA and insulin for their treatment. The majority (76.45) had < 3 drugs prescribed for their treatment each day, while 52.9% showed non-compliance as they reported that they had no money to buy medicines for their disease. The majority (60.4%) had fast blood glucose levels within 101–125 mg/dl, and 5.6% of the patients with diabetes-reported disability arising from long-standing and poor control of diabetes mellitus. Furthermore, in our study, 34.0% of the participants had poor social support during the disease, as shown in Table 2.

**Table 2:** Clinical and psychosocial characteristics of patients with diabetes mellitus attending the outpatient department at SINA Clinics [August–September 2022] (*N* = 493).

**Clinical and psychosocial characteristics**	**Co-variables**	**n (%)**
Age at diagnosis	18–30	21 (4.2)
	31–45	109 (22.1)
	45–54	174 (35.2)
	≥55	186 (37.7)
Duration of DM (in year)	1–5	181 (35.9)
	6–10	142 (28.1)
	≥10	166 (32.9)
DM Rx regime	Insulin	7 (1.4)
	Oral hypoglycemic	294 (58.3)
	Insulin plus oral	189 (37.5)
Duration of DM Rx (in year)	1–5	181 (35.9)
	6–10	142 (28.1)
	≥10	166 (32.9)
No. of prescribed medication administered per day	≤ 3	377 (76.4)
	≥4	116 (23.6)
Compliant with medication'	Yes	392 (79.5)
	No	101 (20.4)
Reasons for non-compliance (*n* = 202)	No money to buy it	107 (52.9)
	Drug side effect	53 (26.2)
	Others	42 (20.7)
Ever measured FBG	Yes	362 (73.4)
	No	131 (26.5)
FBG level (in mg/dl)	≤ 100	58 (11.7)
	101–125	298 (60.4)
	≥126	137 (27.7)
DM complication	Yes	127 (25.7)
	No	366 (74.2)
Physical disability	Yes	28 (5.6)
	No	465 (94.4)
Social support	Poor	168 (34.0)
	Intermediate	139 (28.1)
	Strong	186 (37.7)

Depression was prevalent in 30.83 % of diabetes individuals (95% CI: 26.4, 35.2). Of the 493 individuals, 10.1, 7.5, 5.0, and 2.6% met the criteria for mild, moderate, moderately severe, and severe depression, respectively. In this study, living in an urban area, having a fast blood glucose (FBG) level of 126 mg/dl, being physically disabled due to long-standing diabetes mellitus, and having little or poor social support during illness were all related to depression in the bivariate analysis (Table 3). However, being physically disabled (AOR: 4.70, 95%CI = 1.28, 6.17) and having low or poor social support (AOR: 3.41, 95%CI = 1.76, 6.36) were revealed to be independent predictors of depression among diabetic patients in the multivariate analysis (Table 4) In this study, 35.5% of patients had wished to be dead in the past week, while 24.3% f felt that their family would be better off if they were dead. Moreover, 20.3% thought about killing themselves, while 11.8% attempted to kill themselves. Furthermore, we found 15.1% of participants with an acute positive screen (imminent risk identified) who require a STAT safety/full mental health evaluation as shown in Table 5.

**Table 3:** Bivariate analysis of depression among patients with diabetes mellitus attending the outpatient department at SINA Clinics [August–September 2022] (*N* = 493, *n* = 152).

**Variables**	**Co-variables**	**Depression (*n*) yes**	**Depression (*n*) no**	**COR (95% CI)**	***P*-value**
**Residence**	Urban	148	330	2.20 (0.33, 3.68)	0.01
	Rural	4	8	0.48 (0.23, 1.22)	0.10
**FBG level**	≤ 100	16	42	1.10 (0.53, 1.68)	0.13
	101–125	60	238	1.40 (0.53, 2.68)	0.12
	≥126	76	61	2.14 (1.26, 5.12)	0.14
**Social support**	Poor	93	75	3.34 (2.38, 6.52)	0.02
	Intermediate	40	99	1.20 (0.71, 2.90)	0.02
	Strong	19	167	1.40 (0.53, 2.68)	0.12
**Physical disability**	Yes	16	12	3.48 (1.30, 8.26)	0.01
	No	136	429	1.40 (0.53, 2.68)	0.10

**Table 4:** Multivariate analysis of depression among patients with diabetes mellitus attending the outpatient department at SINA Clinics [August–September 2022] (*N* = 493, *n* = 152).

**Variables**	**Co-variables**	**Depression (*n*) yes**	**Depression (*n*) no**	**AOR (95% CI)**	***P*-value**
Residence	Urban	148	330	1.00	
	Rural	4	8	0.43 (0.13, 1.39)	0.71
FBG level	≤ 100	16	42	1.00	
	101–125	60	238	1.40 (0.43, 2.89)	0.64
	≥126	76	61	3.01 (1.03, 5.02)	0.67
Socioeconomic Status	Poor	140	263	4.21 (2.26, 7.36)	0.00
	Intermediate	11	76	1.42 (0.26, 4.26)	
	Rich	1	2	1.00	
Social support	Poor	93	75	3.41 (1.76, 6.36)	0.02
	Intermediate	40	99	1.42 (0.66, 3.08)	0.81
	Strong	19	167	1.00	
Physical disability	Yes	16	12	4.70 (1.28, 6.17)	0.032
	No	136	429	1.00	

**Table 5:** Screening of patients using the Ask Suicide Screening Questionnaire for suicidal ideation among depressed patients.

**Ask suicide screening questions (*****n*** = **152)**		**Yes**	**No**
1	In the past few weeks, have you wished you were dead?	54	98
2	In the past few weeks, have you felt that you or your family would be better off if you were dead?	37	115
3	In the past week, have you been having thoughts about killing yourself?	31	121
4	Have you ever tried to kill yourself?	18	134
5	Are you having thoughts of killing yourself right now?	23	129

If patient answers “No” to all questions 1 through 4, screening is complete (not necessary to ask question #5). No intervention is necessary (Note: Clinical judgment can always override a negative screen). • If patient answers “Yes” to any of questions 1 through 4, or refuses to answer, they are considered a positive screen. Ask question #5 to assess acuity: o “Yes” to question #5 = acute positive screen (imminent risk identified) • Patient requires a STAT safety/full mental health evaluation. Patient cannot leave until evaluated for safety. • Keep patient in sight. Remove all dangerous objects from the room. Alert physician or clinician responsible for patient's care. o “No” to question, #5 = non-acute positive screen (potential risk identified) • Patient requires a brief suicide safety assessment to determine whether a full mental health evaluation is needed. Patient cannot leave until evaluated for safety. • Alert physician or clinician responsible for patient's care.

## Discussion

This study aimed to determine the prevalence and risk factors for depression in individuals with diabetes mellitus at the outpatient department of SINA clinics in an urban slum in Karachi, Pakistan. According to the current study, the prevalence of depression is 30.83% of individuals suffering from diabetes mellitus. The current study's findings were consistent with a cross-sectional investigation conducted in Egypt (33.3%) and Bahrain (35%), respectively (13, 14). The prevalence reported in this study was also higher than that reported by Engidaw et al. (11) and Waitzfelder et al. (7) in their study from Ethiopia and USA, respectively. However, much higher prevalence rates of depression among patients with diabetes were reported from studies undertaken in India (43.4%) and the Islamic Republic of Iran (73.4%), respectively (9, 15).

The disparity might be attributed to disparities in evaluation instruments, healthcare delivery systems, educational status, lifestyle, and social contact. In Egypt, for example, the MADRS screening test was used to assess depression. On the contrary, the Beck Depression Inventory (BDI) was used to measure depression in studies conducted in Iran and Ethiopia. Another cause for the disparity might be different study conditions and different ethnicity of the individuals taken up for the study.

On the other hand, the findings of this research were more significant than those done in Malaysia, the University of Gondar diabetes clinic, and the Black Lion Specialized Hospital, with 12.3, 15.4, and 13%, respectively (16–18). One reason for the disparity might be the varied healthcare systems and the fact that the PHQ-9 cutoff score is 5. Furthermore, all the initial research was done in specialist hospitals. Those treated in a specialty hospital may receive more thorough care due to ample qualified human resources. In our setting, such high-quality health facilities and resources were not available.

We also investigated the risk factors for depression in people with diabetes mellitus. As a result, diabetic patients with poor social support were 3.34 times more likely to develop depression than diabetic patients with good social support. This finding was consistent with research done in Ethiopia at the Felege-Hiwot referral and Black Lion Specialized Hospitals (19, 20). This might be because social isolation decreases social support, which can have a negative impact on physical and mental health. Diabetes therapy may be delayed due to a lack of social support. If treatment is delayed, the patient will show signs of potential signs of diabetes complications, which predispose the patient to various psychological problems such as depression (21). Patients with diabetes who were physically impaired were 4.7 times as likely than non-disabled individuals to be depressed. The cause might be that physical infirmity leads to unemployment, fewer educational possibilities, and social contacts, all of which may predispose the patient to depression. Another cause might be that persons with physical disabilities do not get enough physical activity (22).

Furthermore, it was found that those who were socioeconomically poor were 4.2 times more at risk of having depression than those who were socioeconomically stable. Earlier studies have also reported that low levels of household income are linked to a variety of lifelong mental illnesses and suicide attempts, and a decrease in household income is linked to an increased risk of incident mental disorders (23).

Furthermore, the suicidal ideation among depressed patients with diabetes was analyzed using the Ask Suicide Screening Questionnaire for suicidal ideation, in which it was found that 20.39% had suicidal ideation thoughts. Suicidal ideation and attempt rates have been reported as high as 26.4 and 13.3%, respectively (24, 25). Some investigations on suicidal ideation and attempts in patients with diabetes found that suicidal risk increases in diabetics (26, 27). However, two investigations indicated that patients with diabetes had a lower risk of suicidal thoughts than healthy controls and patients with other medical disorders (28, 29). Depression has been identified as the most frequent psychological illness among people with diabetes who attempted suicide (30).

## Strengths and limitations of the study

The study's strengths were the use of a relatively large sample size with a reasonable response rate and the use of validated techniques. The current study also has several significant limitations that should be considered when interpreting the findings. Because this study was done in health institutions, the findings may not accurately reflect the depression of all patients with diabetes. The study's cross-sectional design does not prove a definitive cause-and-effect relationship.

## Conclusion

Depression was connected with having type II diabetes, inadequate family and community support, and being physically impaired. Clinicians must prioritize patients with diabetes who are physically disabled and have limited social support. Early diagnosis and treatment of depressive symptoms as a standard component of diabetes care is suggested for doctors who deal closely with patients with diabetes.

## Data availability statement

The original contributions presented in the study are included in the article/supplementary material, further inquiries can be directed to the corresponding author.

## Ethics statement

The studies involving human participants were reviewed and approved by SINA-ETHICAL REVIEW BOARD (SINA-ERB). Written informed consent for participation was not required for this study in accordance with the national legislation and the institutional requirements.

## Author contributions

HS proposed the study design and study concept and prepared the manuscript. SJ helped in the literature review and finalized the results. TS and HN gathered and sorted out the data. SS was involved in data analysis and the result interpretation. SJ and ZJ supervised the overall project and reviewed the final version of the manuscript. All authors contributed to the article and approved the submitted version.
